# Efficient Generation of Permutationally Invariant Potential Energy Surfaces for Large Molecules

**DOI:** 10.1021/acs.jctc.0c00001

**Published:** 2020-03-26

**Authors:** Riccardo Conte, Chen Qu, Paul L. Houston, Joel M. Bowman

**Affiliations:** † Dipartimento di Chimica, Università Degli Studi di Milano, via Golgi 19, 20133 Milano, Italy; ‡ Department of Chemistry & Biochemistry, University of Maryland, College Park, Maryland 20742, United States; § Department of Chemistry and Chemical Biology, Cornell University, Ithaca, New York 14853, United States; ∥ Department of Chemistry and Biochemistry, Georgia Institute of Technology, Atlanta, Georgia 30332, United States; ⊥ Department of Chemistry and Cherry L. Emerson Center for Scientific Computation, Emory University, Atlanta, Georgia 30322, United States

## Abstract

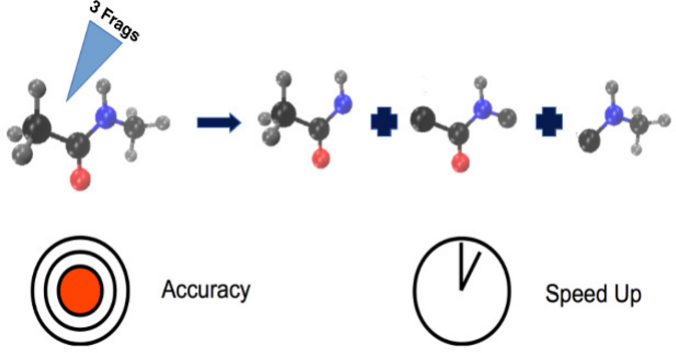

An efficient method is described for generating a fragmented, permutationally invariant polynomial basis to fit electronic energies and, if available, gradients for large molecules. The method presented rests on the fragmentation of a large molecule into any number of fragments while maintaining the permutational invariance and uniqueness of the polynomials. The new approach improves on a previous one reported by Qu and Bowman by avoiding repetition of polynomials in the fitting basis set and speeding up gradient evaluations while keeping the accuracy of the PES. The method is demonstrated for CH_3_–NH–CO–CH_3_ (*N*-methylacetamide) and NH_2_–CH_2_–COOH (glycine).

## 1 Introduction

Developing high-dimensional, *ab initio*-based potential energy surfaces (PESs) is a long-term and currently very active area of theoretical and computational research. In the past 15 years, significant progress has been made in the development of nonparametric, machine learning approaches to fit large data sets of electronic energies for polyatomic molecules and clusters.^1−15^ These approaches include several that have been extensively applied to date. They are permutationally invariant polynomials (PIPs),^1,14^ Neural Networks (NN),^3−6,16−19^ NN with PIP inputs,^10−13,20,21^ Gaussian Process regression (GPR),^8,15,22^ and GPR with PIP inputs.^23^ There is a major motivation to extend these methods to large molecules of interest in chemistry, biochemistry, and materials science. However, there are significant challenges in doing this for the various approaches.

Our group has developed the PIP approach over the last 15 years to represent high dimensional PESs of molecules and molecular clusters with numerous applications.^1,14,24−26^ This method makes use of Morse variables, which are transformed internuclear distances. In 2003 the approach was first applied to the CH_5_^+^ cation to construct a global PES that is invariant with respect to the 120 possible permutations of the five equivalent H atoms.^27^ Generally the data sets consist of 10^4^–10^5^ scattered electronic energies, typically obtained at the CCSD(T) level of theory. Here “scattered” means nongrid based energies; typically, the data are from a number of low-level direct dynamics trajectory calculations run at different total energies and in some instances from very different initial configurations. More details can be found elsewhere.^1^ This approach has been applied to obtain PESs for more than 50 molecules, including reactive systems, and molecular clusters.^14^ Of particular interest to this paper, there are PESs for 7, 8, 9, and 10 atom systems, e.g., CH_3_CHO, with many minima and saddle points,^28^ CH_3_CHOO,^29^ malonaldehyde,^30^ and the ten-atom formic acid dimer,^31^ respectively.

There are bottlenecks for the PIP methods as the molecular size increases, and these have been discussed previously.^32^ To recap these briefly, the inputs are the values of all the Morse variables and the number of variables grows as order *N*^2^. For the PIP-NN approach the input is the minimum number of PIPs to correctly describe the symmetry of the molecule. This number is larger than the number of Morse variables and grows rapidly with the number of atoms. The growth in the size of PIP bases with the number of atoms depends on both the total polynomial order and the order of symmetric group.^1^ As noted above, the PIP approach has been applied for molecules with as many as 10 atoms and this value has been cited in the literature as the practical limit for the PIP, while the PIP-NN approach has been applied to the seven atom OH + CH_4_ reaction.^33^

The “ten-atom limit” using the PIP approach was broken for the 12-atom *trans*-*N*-methylacetamide (*trans*-NMA).^32^ The major point of that paper, which is preliminary to the present one, was to describe a fragmented PIP approach able to extend the PIP method to larger molecules. As an aside, we mention that the 10-atom limit was also just exceeded using PIPs in the construction of an interaction surface for the CH_4_–H_2_O–H_2_O system,^34^ and in a calculation of anharmonic rovibrational partition functions including torsional motion.^35^

As for ML approaches, we note that they have been extensively developed and applied mainly in the context of materials chemical physics.^4,7,36^ These approaches have in common the development of either a NN or GPR of the atomic energy of each atom. Thus, these methods have been extended to large numbers (e.g., hundreds) of atoms at the cost of a large number of NN or GP evaluations. A recent paper comparing important aspects of atom-based NN and PIP-NN approaches for several molecules^37^ indicates that for “small” molecules the PIP approach is probably the preferred one.

There is, we reasoned,^32,38^ a regime for molecules with more than 10 atoms and probably less than hundreds of atoms where the PIP approach could be extended. The basic observation that enables this extension is that Morse variables go asymptotically to zero at large internuclear distances. Thus, for large molecules many Morse variables are essentially zero and thus any PIP basis function containing these variables is zero and can be dropped from the total basis. This observation allows one to fragment the larger target molecule into smaller moieties for which PIP basis sets can be generated efficiently.^32,38^ The pruning approach is indeed successful and it was used with recent, extended MSA software that includes gradient data for fits.^39^ However, several issues with the algorithm used were noted. These included redundant terms in the basis and also the cost of evaluating gradients, as described in detail below.

New software, described in this paper, solves these two problems. To begin, recall that the PIP approach represents the potential as follows, using compact notation:
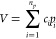
1where *c*_*i*_ are coefficients, *p*_*i*_ are permutationally invariant polynomials, denoted as PIPs (the basis set), and *n*_*p*_ is the total number of polynomials for a given maximum polynomial order. The *p*_*i*_ are typically functions of Morse variables, which themselves are functions of the interatomic distances, *r*_α,β_ (by the usual exponential relationship exp(−*r*_α,β_/λ), where λ is commonly chosen to be equal to 2 bohr). Following the practice of the MSA software, Morse variables are in this work denoted by *x*_*l*_. We further stipulate that the basis functions should be chosen to maintain permutational invariance among identical atoms, or at least some of them. The linear coefficients are obtained using standard least-squares fits to large data sets of electronic energies at “scattered” geometries.

In standard PIP approaches, the computational issue arises when the basis set for the parent molecule is completely unwieldy, too big to be useful in practical terms, either because calculating the proper basis set takes too long or because the number of coefficients is so large that the least-squares optimization becomes problematic. The size of the basis varies in a complicated and generally nonlinear way with respect to the number of Morse variables, the maximum polynomial order, and the order of the symmetric group.^1^ This growth in the size of the PIP basis is the basic consideration in stating the 10-atom limit for the approach.

However, as noted above, the fragmented basis approach is an effective way to break this limit. Clearly, by fragmenting a parent molecule into groups of smaller molecular moieties the basis for each smaller moiety can be calculated rapidly and then combined with those of other fragments to provide a compact and hopefully still precise representation of the potential energy.^32^ To be specific, consider a simple example of a five-atom molecule with atoms labeled as 1–5 and a scheme in which the molecule is fragmented into three fragments, e.g., {1, 2, 3}, {2, 3, 4}, {3, 4, 5}. In this three-fragment scheme the potential is given compactly by
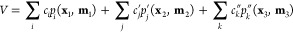
2where {*p*}, {*p*′}, and {*p*″} are PIP bases for the *n*th fragment, (*n* = 1, 2, 3), {*c*}, {*c*′}, {*c*″} are the corresponding linear coefficients, **x**_*n*_ represent the set of corresponding Morse variables, and **m**_*n*_ indicate a set of monomials built from the Morse variables. Morse variables between atoms 1 and 4, atoms 1 and 5, and atoms 2 and 5 are assumed to be zero and hence not in the fragmented bases. In this example there are some Morse variables in common among the fragments, and it should be clear that there are indeed some redundant basis functions in this expression in terms of common Morse variables.

These issues were pointed out previously;^32,38^ however, they were not “fatal” ones, because the linear least-squares method used was able to deal with a modest number of identical basis functions. Nevertheless, there is compelling motivation to eliminate these redundant basis functions and thereby reduce the size of the basis. We do note the redundant-term issue is similar to an issue identified earlier for developing PIP representations of interaction potentials that should rigorously vanish in asymptotic regions where there is no interfragment interaction. In that case the issue concerned basis functions involving Morse variables of fragments that do not go to zero at large internuclear distances where rigorously there is no interfragment interaction. An effective “empirical” pruning procedure was then employed to eliminate such basis functions and applied to several systems.^34,40,41^

An “empirical” pruning approach is also developed here as a postprocessing task performed on the standard complete PIP bases of the fragments. The entire computational approach is described in detail in the next section followed by illustrations for *N*-methylacetamide (NMA) and glycine. These are not large molecules; however, they serve to test the effectiveness of various fragmentation approaches, as full PIP basis fits can be done for these molecules. NMA does contain a low-barrier methyl rotor and so this example should be relevant to many large molecules with methyl rotors. We will make further comments and suggest some guiding principles on how fragmentation of the basis might be applied to challenging molecules that undergo isomerization and/or unimolecular breakup.

## 2 Computational Methods

Following the work of Xie and Bowman,^42^ Xie has provided software^43^ based on a monomial symmetrization approach (MSA) that generates the permutationally invariant basis set for many permutational symmetries and polynomial orders. The monomials are functions of the Morse variables and are combined to form permutationally invariant polynomials up to a select order in a clever and efficient system that allows each polynomial to be recursively determined from simple sums of products of previously calculated monomials or polynomials. In this way, the specific polynomials *p*_*i*_ = *p*_*i*_(**x**, **m**) in eqs 1 and 2 are calculated from the monomials, **m**, as well as from other polynomials *p*_*j*_ with *j* < *i* rather than from the repetitive and more complicated evaluation of functions of the Morse variables, **x**. For example, the MSA program may determine that *p*_128_ may be written as *p*_128_ = *p*_32_*p*_61_ + *m*_4_. At the time when *p*_128_ is evaluated, the components *p*_32_, *p*_61_, and *m*_4_ have already been calculated, making the computation of *p*_128_ very efficient. A Perl program, postemsa.pl, is used to convert the recursion relationships determined by the MSA software to a Fortran program bemsa*.f90, where * stands for the permutational symmetry numbers and the desired fit order. In addition to subroutines that efficiently calculate the monomial and polynomial basis function values, the Fortran program also includes a function that uses these to calculate the energy based on a list of coefficients and the inputs of Cartesian coordinates for each atom rigorously entered in the order of the chosen permutational symmetry. The coefficients are determined by using the bemsa*.f90 program to fit a number of known energies for different geometries. (This software was recently extended to obtain gradient of the energy as well.^44^) As mentioned in the Introduction, it is often quite time-consuming to generate the MSA output, particularly for large molecules. For example, using a single Intel xeon16 (1.2 GHz) processor, our calculation of a basis for 10-atom glycine, with maximum total polynomial order of 4 took more than 12 days. This basis contains 46 654 polynomials. Fragmentation of the parent compound can speed up this process substantially and provide an efficient basis set that allows for faster energy evaluation without sacrificing accuracy. This has been demonstrated already.^32,38^ However, as anticipated, an issue of replicated basis functions was noted.

Owing to the efficient recursive algorithm in the MSA software it is not trivial to identify and eliminate replicated basis functions, and then reorder the basis. We do that here with new Mathematica software.^45^ This software starts with the bemsa*.f90 files created by the MSA software for each of the fragments and generates a list of the unique Morse variables, monomials, and polynomials (here denoted collectively as “(**x**, **m**, **p**)”) while maintaining permutational invariance. Mathematica was chosen because it has both the tools to do complicated string manipulations and the ability to turn strings of texts into commands that can be immediately evaluated. Thus, the MSA Fortran program can be read as a string, the strings can be converted to Mathematica format, and the Mathematica format can then be evaluated. Conversely, once a new recursive scheme has been developed combining the fragment basis sets and eliminating duplicate variables, it can then be converted to a text string, translated into Fortran format, and output as the Fortran program (DuplicatesDeletedbemsa.f90). Additionally, if desired, the new recursive scheme can efficiently incorporate fitting of gradients if these are available. In summary, there are two main goals of the Mathematica program: (1) to increase efficiency by eliminating duplicated Morse variables, monomials and polynomials in the basis set of the ensemble of fragments and (2) to provide an efficient method for calculating derivatives of the energy as a function of the Cartesian coordinates. We outline the methods for achieving these goals next.

As for point 1, we developed two strategies for deleting duplicate Morse variables, monomials, and polynomials: a sequential method, and a pairwise method. Further details of the two methods are included in section S1 of the Supporting Information.

The second goal of our program is to provide an efficient approach for calculating derivatives of the energy with respect to all Cartesian coordinates of all atoms at the geometry corresponding to the energy. The straightforward method for doing this is relatively easy but inefficient; that is, one mechanically takes the derivatives of all monomials (assigned to variables d*m*_*i*_) and polynomials (assigned to variables d*p*_*i*_) and uses these to evaluate the derivative of the potential energy with respect to each of the Cartesian coordinates of the atoms. This is the method used successfully by Qu and Bowman^32^ and by Nandi, Qu, and Bowman.^38^ One main reason why the inefficiency comes about is that, while there are up to *N*(*N* – 1)/2 interatomic distances (where *N* is the number of atoms in the parent), the coordinates of a given atom occur only in *N* – 1 of these. Thus, most of the derivatives with respect to a Cartesian coordinate of a particular atom are zero; there is no reason to calculate them, especially because each calculation involves evaluating many members of the basis set. Specifically, if *a*_*j*,*k*_ with *j* = 1, *N* and *k* = 1, 3 represents the *k*th Cartesian component of the *j*th atom, then the derivative of eq 1 can be written as
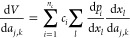
3where *l* enumerates the Morse variables. If the Morse variable *x*_*l*_ do not depend on *a*_*j*,*k*_, then  is zero and the *l*th contribution to the second sum does not need to be evaluated. Our program avoids these “zero” calculations by branching to a separate calculation of eq 3 for each atom *j*. These separate calculations are initiated by setting all variables d*p*_*i*_ to zero and then calculating only the terms in the second sum for which  for at least one combination of *l*, *j*, and *k*.

Prior to running the Mathematica program, one needs to assign a numbering scheme to the parent molecule; each atom should receive an integer number between 1 and *N*, the total number of atoms in the parent. The ordering of the atoms can be whatever is convenient, but, once established, that numbering needs to be consistent throughout the program and the simulations. The atoms of the fragments need to have the same numbers as they do in the parent, and the order in which the geometries are entered as input to the final Fortran program for calculating the energies and derivatives must be the same as the numerical order of the atoms. The inputs and outputs of the Mathematica program are described in section S2 of the Supporting Information. A Mathematica Notebook showing examples and a Wolfram Language Function set are also available in the Supporting Information

An important point concerns the selection of the permutational symmetry for the fragments; it can rarely be the highest symmetry for the fragment considered individually. Consider a parent molecule with a fragment that has among others one atom A and two atoms B. Considered by itself, this fragment would produce a monomial in which the two AB bonds were treated as symmetrical and interchangeable. Now consider another fragment of the same parent that has among others the same atom A and one of the atoms B. Considered by itself, this fragment would produce a monomial that treats the AB bond without any symmetry; it would not be interchangeable with any other bond. When these two fragments are combined, the composite basis set could not have permutational symmetry concerning the two B atoms. This leads to the following rule: In order to maintain permutational invariance for the final basis set, atoms that are assigned to permute with one another must appear together whenever they appear in any of the fragments.

We end this section by noting that once one has a fit, generated either from the original MSA software or from the new MSA_FRAG software here introduced, it is relatively easy to either add or subtract polynomials in order to increase the accuracy or increase the speed, respectively. The principle for selecting the polynomials is as follows. First, determine the maximum value of all the Morse variables among all the geometries in the *ab initio* data set. In order to add polynomials for increased accuracy, consider all those polynomials already used in the existing fit, make all combinations of these up to the desired order, discard those combinations already in use, evaluate all the new combinations using the maximum values of the Morse variables, and add those combinations to the basis set that have the largest values. In order to delete polynomials for increased speed, evaluate all the existing polynomials using the maximum values of the Morse variables and discard as many as desired starting from the lowest and moving to the highest. In the latter case, the new set of polynomials will need to be examined for (new) duplicates, and in both cases the polynomials and monomials will need to be renumbered. The new set will retain any permutational invariance that was present in the original set. It is important to point out that for large molecules it is typically not possible to generate the MSA output for the parent compound; one needs to fragment the molecule to get the number of original polynomials (coefficients) down to a manageable number.

## 3 Results and Discussion

The fragmentation approach^32^ is relatively new, so that at this point determination of the best fragmentation approach is still an art rather than a science. One objective of this paper is to make the approach easier for others to use so that collective intuition can be developed. Nonetheless, a few principles are clear, although these involve more mathematical intuition than chemical. The goal is to include those Morse variables with large values and to exclude those that will always have small values. Recall that the Morse variables have values between 1 and 0 and fall off exponentially with the distance between the two atoms. First, we note that there is no particular need for the fragments to be connected in a way that reflects the parent molecule; what one seeks is to have those atoms that are close to one another (and whose pairs have relatively large Morse values) included in at least one fragment. Morse variables involving distant atoms will be small, and these can be omitted by never including the relevant atoms in the same fragment. We note, however, that even if all Morse variables are included, the number of monomials and polynomials in the fragmented, permutationally invariant basis set will still be smaller in fragmentation than in the parent compound because the fragmentation reduces the cross terms between these variables. If one desires particular cross terms, then the relevant atoms for the Morse variables should be included in at least one fragment. Finally, fragments will often have atoms in common, atoms that have large Morse variables with others in each fragment, even though other atoms between the fragments may not have large Morse values. The overlap is often needed and should not be shunned. The Delete Duplicates program ensures that Morse variables, monomials, and polynomials based on the same pair of atoms are not included more than once.

### 3.1 CH_3_–NH–CO–CH_3_ (*N*-methyl Acetamide, NMA)

**Figure 1:**
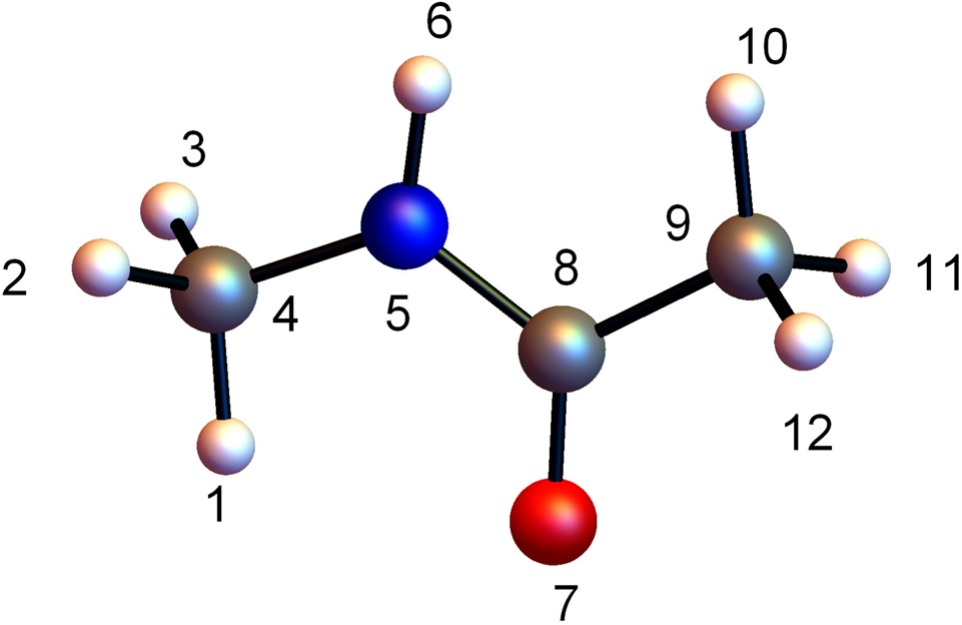
Numbering scheme used in text for NMA. H, C, O, and N atoms are white, gray, red, and blue, respectively.

In order to test the outcomes of our program we chose to calculate results for CH_3_–NH–CO–CH_3_ (NMA) because this molecule had already been studied by Qu and Bowman and results on accuracy were available from their work.^32^ They reported results for the full parent molecule, for two fragments, CH_3_–NHC–CO and C–NH–CO–CH_3_, and for three fragments, CH_3_–NH–C, N–CO–CH_3_, and C–NH–CO–C. We also here report results for a five-fragment calculation where the fragments are CH_3_–NH–C, N–CO–CH_3_, C–NH–CO–C, and H_3_–O–C, C–H–H_3_. We followed the same numbering scheme, shown in Figure 1, and used exactly the same *ab initio* data set for fitting as Qu and Bowman.^32^ The data set consisted of 3000 energies and 3000 × 36 = 108 000 Cartesian gradient components. Both energies and gradients in the data set have been fitted.

By the example of NMA, we also hope to clarify issues concerning inputs 3 and 4 (described in Section S2 of the Supporting Information) by considering in detail the permutational symmetry and atom entries for the two-fragment and three-fragment cases. In the two-fragment case, we have CH_3_NHCOC and CNHCOCH_3_. In order to maintain permutational invariance for the composite fragmented system, the H atoms on either methyl can be assigned to permute with one another because they always appear together, but those on the two methyls may not be assigned to permute between one another because one methyl is missing from each fragment. By our rule (see above), it would be possible to allow the three carbons in the set of common atoms to permute with one another because they appear in both fragments, but we may not allow the H on the N to permute with any other H atoms because in each fragment some of the other H atoms are missing. Following Qu and Bowman, we choose to ignore the carbon permutation, so that only the three H atoms on the methyl group of either fragment permute with one another; also there is no permutation of H atoms between the two ends. The permutational symmetry for each fragment is thus {3, 1, 1, 1, 1, 1, 1}, and a possible atom listing is {1, 2, 3, 4, 5, 6, 7, 8, 9} for the first fragment and {10, 11, 12, 4, 5, 6, 7, 8, 9} for the second fragment. The notation here indicates that in the first fragment, for example, atoms 1, 2, and 3, the hydrogens on the left-hand methyl, permute among one another and that none of the other atoms permutes. Note that there are many possible atom listings because the ordering within one symmetry element is irrelevant and the ordering between groups of the same symmetry is also irrelevant. Thus, for the first fragment the atom listing {2, 1, 3, 5, 9, 7, 4, 8, 6} would also work. The two-fragment case essentially assumes that there is no interaction between the hydrogens on one end and those on the other; thus 9 Morse variables are neglected.

For the three-fragment case, we have CH_3_–NH–C, N–CO–CH_3_, and C–NH–CO–C. In order to maintain permutational invariance for the composite fragmented system, we may allow the H atoms on either methyl to permute with one another, since they always appear together when they are present, but no permutation may be assigned between the H atoms on opposite methyls, as in the two-fragment case. The carbons may not be allowed to permute, because the first fragment has the left and middle one, which do not appear in fragment 2, while the second fragment has the middle and right one, which do not appear in fragment 1. The H on the NH is similarly not assigned to permute with any other H atoms, because they are not present, for example, in fragment 3. The permutational symmetry for the first and second fragments is {3, 1, 1, 1, 1}, with atom assignments of, for example, {1, 2, 3, 4, 5, 6, 8} and {10, 11, 12, 5, 7, 8, 9}, while the third fragment has symmetry {1, 1, 1, 1, 1, 1} with an atom assignment of, for example, {4, 5, 6, 7, 8, 9}.

We note that Nandi et al.^46^ have recently extended the previous study of the NMA PES to the cis isomer and transition states. They used the above two-fragment case and a different three-fragment case which consists of the previous and present two-fragment case plus nonoverlapping fragments consisting of the three H atoms on each methyl group. This six-atom fragment basis was denoted as {3, 3}. This work demonstrates that the fragmentation approach can describe isomerization.

Table 1 shows the results of our work in comparison to those of the previous study.^32^ For the full molecule there are 66 Morse variables and 8040 polynomials. The root-mean-square errors for the energies and gradients, as compared to the *ab initio* data set are provided. The times listed are those for evaluating 3000 energies and 3000 × 36 gradients on an intel i7 (2.7 GHz) processor. The two-fragment results are given in columns 3 and 4. The results from the previous work^32^ take about half the evaluation time as the full set. The results of our basis set and derivative evaluation method provide exactly the same RMSE and RMSG but take only about 75% as much time as the previous two-fragment calculation. There are 57 Morse variables in both cases, but there are 13.5% fewer polynomials in our basis set due to the deletion of duplicates. Similarly, for the three-fragment case the RMSE and RMSG results are identical, but the size of our basis set is 8.5% smaller and the time required is 24% lower. The five-fragment result provides a basis set that is between that of the two-fragment and three-fragment ones, with correspondingly intermediate RMS error values and timings.

**Table 1:** NMA Results (RMSE in cm^–1^; RMSG in cm^–1^/bohr)

	ref (32)	this work	ref (32)	this work	ref (32)	this work
fit order	3	3	3	3	3	3
frags	full	2	2	3	3	5
Morse vars	66	57	57	45	45	57
Polys	8040	5240	6056	1806	1974	1936
RMSE	26.8	34.3	34.3	148.9	148.9	93.1
RMSG	54.7	67.4	67.4	171.8	171.9	141.8
time/s	6.611	2.450	3.281	0.830	1.056	0.914

It appears from these tests that our program has achieved the desired results of deleting duplicates and accelerating the calculation. In all cases, our program tested the results to be sure that there were no remaining duplicated monomials or polynomials, and it also tested to confirm that the basis set maintained permutational invariance.

### 3.2 NH_2_–CH_2_–COOH (Glycine)

Glycine is the smallest amino acid and one of the building blocks of proteins. Its biological importance as a precursor of life has triggered the search for it in the interstellar medium through exprimental spectroscopic investigations which require theoretical validation.^47^ For this reason, different theoretical approaches have been undertaken in the attempt to clarify glycine spectral features and describe accurately its elusive isomers and complicated potential energy surface. The surface and frequencies of vibration have been described in several ways ranging from reduced-dimensional models^48^ to harmonic approximations^49^ and from semiempirical electronic structure methods^50^ to static and dynamical *ab initio* approaches.^51−53^ In spite of all the interest around this amino acid, to best of our knowledge an accurate (and possibly permutationally invariant) potential energy surface is missing, probably due to its topological complexity and the nontrivial number of atoms to deal with. In the following we will contribute to partially fill this gap by focusing on the global minimum well.

**Figure 2:**
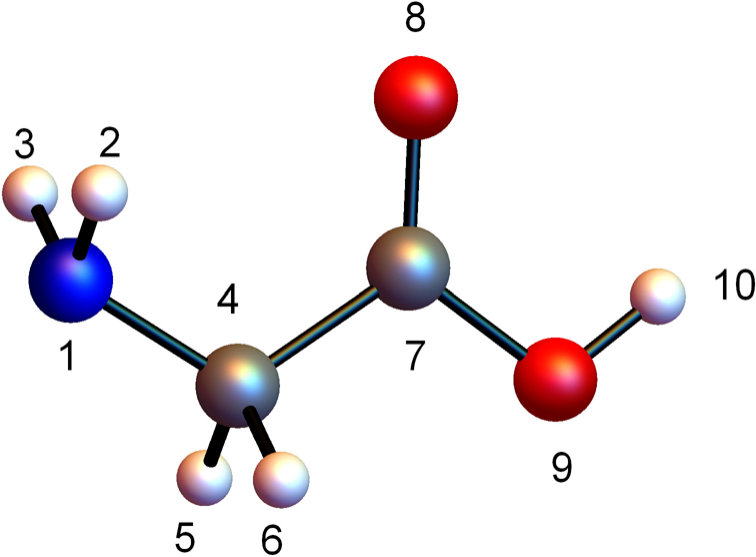
Numbering scheme used in the text for Glycine. H, C, O, and N atoms are white, gray, red, and blue, respectively.

The glycine minimum energy structure and the (arbitrary) numbering used here are shown in Figure 2. The equilibrium geometry is of *C*_*s*_ symmetry and often conformationally referred to as an *all-trans* (ttt) structure,^54,55^ with reference respectively to the relative positions of hydrogens (atoms 2–5) in the H–N–C-H bond chain (2–1–4–5), NH_2_ (1–2–3), and OH (9–10) groups with respect to the C–C bond (4–7), and the hydroxy hydrogen (10) relative to the C atom (4) with respect to the C–O bond involving the hydroxy group (7–9).

We considered six potential energy surfaces for glycine, three with basis set polynomials to third-order and three with basis set polynomials to fourth order. In both fourth-order and third-order potential energy surfaces we considered basis sets corresponding to the complete molecule, to two fragments, and to three fragments. For each surface, the minimum energy geometry was determined by a gradient method and the harmonic vibrational frequencies were calculated through diagonalization of the mass-scaled Hessian matrix of the potential at the equilibrium geometry.

**Figure 3:**
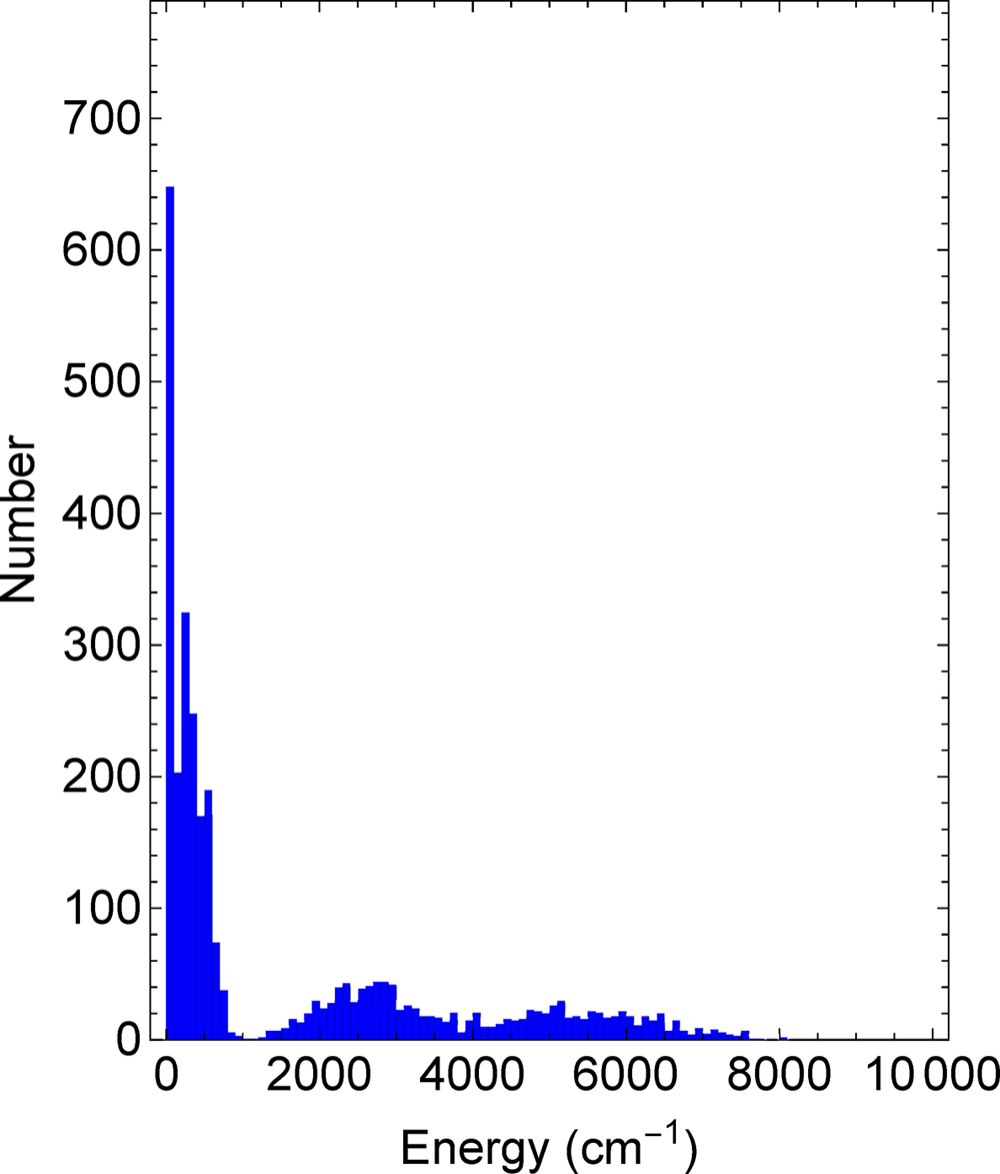
Histogram of energies for geometries used as the data set for glycine. The bin size for the abscissa is 100 cm^–1^. Most energies are for configurations close to that of the global minimum.

The data set used for fitting the surfaces consists of 3100 energies and 3100 × 30 = 93 000 gradient components. Three thousand geometries were chosen from *ab initio* molecular dynamics simulations at energies ≤10 000 cm^–1^, while 100 geometries were chosen randomly on a grid centered at the temporary minimum located on the surface fitted to the preliminary 3000 geometries. A histogram of the 3100 energies along with the approximate energies of the minimum energy structure, the low-energy isomers, and the transition states between them is shown in Figure 3. The molecular dynamics was performed under conditions similar to those used previously for NMA,^32^ and the Molpro calculations were performed using DFT with hybrid B3LYP functional and Dunning’s aug-cc-pVDZ basis set; both energies and gradients were obtained. The same Molpro software was used to get the *ab initio* minimum energy geometry, its energy, the set of gradients, and the benchmark harmonic vibrational frequencies.

For the surfaces corresponding to the nonfragmented molecule, we took the symmetry basis set to be {2, 2, 2, 1, 1, 1, 1}, allowing the H atoms on NH_2_ to permute with one another, the H atoms on CH_2_ to permute with one another, and the two O atoms to permute with one another. We omitted the carbon atom permutations and permutation of the COOH hydrogen in order to be consistent with the fragmentation symmetries to follow. In the two-fragment cases, the fragments are NH_2_–CH_2_ and CH_2_–COOH. The H atoms on CH_2_ may permute with one another, because when they appear, they always appear together. Similarly, the H atoms on NH_2_ may permute with one another for the same reason, but the H atoms on NH_2_ may not be assigned to permute with those on CH_2_ because the NH_2_ atoms do not appear in fragment 2, whereas the CH_2_ ones do. The COOH H atom may not be allowed to permute with the H atoms on either the CH_2_ or the NH_2_ because the second fragment lacks the NH_2_ and the first fragment lacks the COOH. The two oxygens, when they appear, always appear together, so they may be allowed to permute. The two carbon atoms may not be assigned to permute because only one of them appears in each fragment. The permutational symmetry of the first fragment is {2, 2, 1, 1} with an atom assignment, for example, of {2, 3, 5, 6, 1, 4}. The permutational symmetry of the second fragment is {2, 2, 1, 1, 1} with an atom assignment, for example, of {5, 6, 8, 9, 4, 7, 10}.

In the three-fragment cases, we add the fragment NH_2_–COOH to the two from the two-fragment case. Again, the H atoms on NH_2_ may be allowed to permute with one another, the H atoms on the CH_2_ may be allowed to permute with one another, and the O atoms of COOH may be allowed to permute with one another. The permutational symmetry of the third fragment is {2, 2, 1, 1, 1} with an atom assignment, for example, of {2, 3, 8, 9, 1, 7, 10}; the permutation symmetries and atom assignments for the first two of the three fragments remains unchanged from the two-fragment case.

Table 2 shows the results of our glycine calculations. The RMS values for energies and gradients, as compared to those of the fitting data set, are listed. Vibrational frequencies were calculated for the minimum energy structure of each surface. The comparison of these frequencies to those determined by Molpro for the minimum-energy structure is given by the Mean Absolute Error (MAE) row, based on the 24 frequencies calculated. The RMS values were used for the energies and gradients because these are determined by least-squares methods. We used MAE values for the vibrational frequencies because these do not over emphasize a few worse matches. The vibrational frequencies for the *ab initio* surface, for the third-order full molecule surface, and for the third-order, 3-fragment surface are provided in Table 3.

**Table 2:** Glycine Results (RMSE and MAEvib in cm^–1^; RMSG in cm^–1^/bohr)

fit order	3	3	3	4	4	4
frags	full	2	3	full	2	3
Morse vars	45	33	45	45	33	45
Polys	4683	1022	1704	46654	5348	9337
RMSE	20.1	131.6	82.9	7.5	109.3	30.8
RMSG	59.6	354.7	189.3	1.3	257.6	77.2
MAEvib	11.1	31.2	17.5	7.2	27.3	13.6
time/s	2.193	0.408	0.708	23.869	2.192	3.982

**Table 3:** Glycine Results: Vibrational Frequencies for the *ab Initio* Frequencies for the Glycine Global Minimum Provided by Molpro Compared to Those for the Third-Order, Full Molecule, and the Third-Order, Three-Fragment Surfaces (All Energies in cm^–1^)

mode	*ab initio*	third-order, full mol	third-order, three-frag
1	61.7	100.1	34.7
2	211.6	231.2	209.9
3	256.0	260.6	238.6
4	461.3	466.1	459.3
5	512.3	526.0	531.6
6	630.2	631.6	574.2
7	648.4	647.7	641.6
8	816.9	820.6	810.7
9	908.3	906.5	898.5
10	912.2	918.4	942.2
11	1122.6	1124.4	1129.3
12	1160.9	1161.0	1148.4
13	1176.2	1178.2	1158.1
14	1297.8	1291.0	1288.2
15	1371.8	1370.1	1357.7
16	1385.0	1379.0	1374.2
17	1437.3	1444.4	1443.7
18	1656.6	1664.2	1674.6
19	1803.6	1793.3	1787.7
20	3045.9	3037.3	3017.7
21	3083.7	3041.3	3058.7
22	3493.8	3466.4	3457.3
23	3567.1	3547.5	3540.6
24	3735.2	3704.1	3719.5

As expected, the fourth-order, full molecule calculation (Table 2, column 5) not only provides the most accurate results but also takes the most time for the evaluation of 3100 energies and 3100 × 30 gradients (compare across the last row). The third-order, full molecule fit is more than ten times faster with only modest loss of accuracy.

The advantages of fragmentation in glycine (10 atoms) are not as prominent as those in NMA (12 atoms) partly because the full molecule basis set can still be calculated reasonably rapidly, at least at third order. However, if one were to consider a somewhat larger amino acid or even a small peptide, it would be impractical to calculate the basis for the molecule without fragmentation. One aim of research in the future should be to see how fragmentation might help in these larger systems.

**Figure 4:**
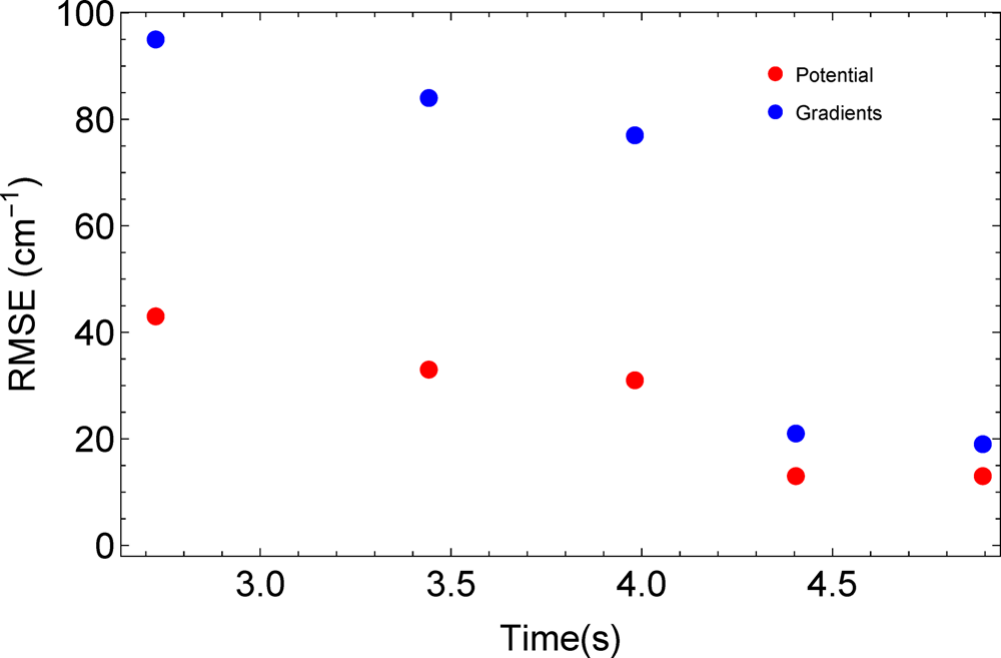
RMSE values for the fourth-order, three-fragment fit for glycine vs the time for calculation of energy and gradients (see text for details). The original fit from the last column of Table 2 is shown by the points near 4.0 s on the abscissa. Points to the left of these are for pruning the number of coefficients back from 9337 to, from the left, 6433 or 7986, or by adding polynomials to give the points to the right, with 10 001 and 11 001 coefficients.

As mentioned in the section on Computational Details, it is possible once a fit has been found to add or delete polynomials to or from the basis set in order to achieve increased accuracy or shorter execution times, respectively. Figure 4 shows an example, based on the fourth-order, three-fragment fit in the last column of Table 2. From the left, the number of coefficients for each set of points at a particular time is 6443, 7986, 9337, 10 001, and 11 001. As can be seen from the figure, the time and accuracy generally both increase with the number of coefficients. One can thus suggest a “figure of merit” for the fit as the product of the average of the RMSE values for the potential or the gradients in cm^–1^ times the time for execution of the computational test in s; the smallest value is desired. This figure of merit for the five cases is, from left to right, 188, 201, 215, 75, and 78, suggesting that the fit with a computational time near 4.4 s and with 10001 coefficients gives the best compromise between accuracy and time. Of course, depending on the application, one might still prefer a faster fit, or perhaps a more accurate one. The adding and pruning of polynomials offers a method to determine a potential fit that has the desired trade-off between accuracy and time.

## 4 Conclusions

The program we have created provides an efficient and practical way to incorporate fragmentation in the choice of basis functions for calculating large-molecule potential energy surfaces. We have tested it against previous (and less efficient) methods for NMA with encouraging results; energies and gradients are exactly reproduced in less time. We have used it to calculate and compare several potential energy surfaces for glycine global minimum conformer. It is practical both because it incorporates all fragments in a single code with relatively simple input parameters and because the outputs include a Fortran program that can be used for fitting the coefficients or using them to calculate energies and gradients.

Glycine was chosen because it is the simplest amino acid, and it is often studied in supramolecular systems of biological interest involving other glycines, water, or hydrogen molecules. For our glycine database we have employed a set of energies up to 10 000 cm^–1^ sampled from trajectories mainly confined within the global minimum well. This choice was driven by the goal of demonstrating the effectiveness of the new software rather than by the necessity to develop a global surface for glycine. The latter would require additional characterization of a number of shallow wells and energetically low-lying transition states, which is not reported here and left for future work. We believe that the present work opens a route to the study of other amino acids, peptides, nucleobases, and more complicated biological structures.

We note that in the examples we have used the increase in speed is modest, but this is because the molecules and fragments are relatively small. For example, there are only 816 duplicated polynomials in the two-fragment NMA case, so the decrease in CPU time is only a bit over 25%. For large molecules with more fragments, the savings in CPU time is expected to be much greater.

More work is needed to develop an understanding of which fragmentation schemes are most effective for different systems. Isomerization has already been shown to be described by the fragmentation approach. Chemical reactions may be more difficult to tackle with fragmentation, as these can involve very large amplitude motion. Of course, the applicability of the approach would be system dependent and we see no reason in principle why it cannot succeed. It is fairly obvious, as shown by both molecular examples here, that when the molecule can bend back on itself it is important to include interactions between the two ends of the molecule. Thus, treating the molecule as a circle and dividing into overlapping fragments around the circle is a good strategy. However, when the molecule is fairly rigid, it is more likely that end-to-end interactions can be neglected, so that dividing the molecule into overlapping fragments along the line and neglecting long-range interactions will be most efficient. It will take some practice and experience to uncover more subtle aspects of the fragmentation method.

## Supporting Information Available

Section S1, the sequential and pairwise methods for deleting duplicates; section S2, inputs and outputs of the Mathematica program; section S3, detailed analysis of fragmentation of a ficitious A_2_BCDEF_2_ molecule using a two-fragment scheme with a single Morse variable in common and table of results for three more complicated fragmentation schemes (PDF)
NMA-FRAG Mathematica programs including a notebook with examples, called DeleteDuplicatesMainProgramAndExamples.nb and a set of functions, called DeleteDuplicates.wl (ZIP)
