# Supplementary material for: Efficient Generation of Permutationally Invariant Potential Energy Surfaces for Large Molecules
Source: J Chem Theory Comput. 2020 Mar 26;16(5):3264–72. doi: 10.1021/acs.jctc.0c00001 (PMC7997398; doi:10.1021/acs.jctc.0c00001)
Supplement: Supplementary file 1 — ct0c00001_si_001.pdf [file ct0c00001_si_001.pdf]

Efficient Generation of Permutationally Invariant Potential Energy Surfaces for Large Molecules Supporting Information

Riccardo Conte,[*,†] Chen Qu,[‡] Paul L. Houston,[*,¶] and Joel M. Bowman[*,§]

†*Dipartimento di Chimica, Università Degli Studi di Milano, via Golgi 19, 20133 Milano, Italy*

‡*Department of Chemistry & Biochemistry, University of Maryland, College Park, Maryland 20742, U.S.A.*

¶*Department of Chemistry and Chemical Biology, Cornell University, Ithaca, New York 14853, U.S.A. and Department of Chemistry and Biochemistry, Georgia Institute of Technology, Atlanta, Georgia 30332, U.S.A*

§*Department of Chemistry and Cherry L. Emerson Center for Scientific Computation, Emory University, Atlanta, Georgia 30322, U.S.A.*

E-mail: riccardo.conte1@unimi.it; plh2@cornell.edu; jmbowma@emory.edu

Phone: +1 404 727-6592

Supporting Information

S1. The sequential and pairwise methods

We developed two strategies for deleting duplicate Morse variables, monomials, and polynomials: a sequential method, and a pairwise method. Each method uses the same technique for identifying the duplicate values of $\mathbf{x}$, $\mathbf{m}$ and $\mathbf{p}$. The way we identify duplicates is numerical. For this reason, on the one hand we have to avoid accidental duplication, i.e. polynomials returning same values even if they are different; on the other hand we do not want to miss any duplicates due to numerical round-off errors. So, our procedure consists of three steps: i) assign a random 16-digit number between 0.1 and 1.0 to each Morse variable. Such a precise assignment is intended to avoid accidental duplication; ii) calculate all monomials and polynomials; iii) multiply the values by 10^8 and compare the integer parts of monomials and polynomials to identify duplicates. This last step is intended to eliminate the possibility ($1/10^8$) of wrong duplicate identifications due to round-off errors. The fast Mathematica command `Position`[*list,pattern*], which gives the position of each *pattern* in the *list*, is used to determine which monomials or polynomials have the same integer part; these are taken to be duplicates and marked for deletion. Since our largest fitting bases have a number of polynomials of the order of 10^4, we estimate that the probability of incorrectly identifying a duplicate is, at worst, on the order of $1/10^4$ or 0.01%.

In the sequential method, a) the `bemsa*.f90` files for all the individual fragments are read in and converted to Mathematica format. For the first fragment, the $(\mathbf{x}, \mathbf{m}, \mathbf{p})$ are taken as provided. b) The following steps are taken for each additional fragment: The numberings of the $(\mathbf{x}, \mathbf{m}, \mathbf{p})$ for the new fragments are augmented by the maximum values of $(\mathbf{x}, \mathbf{m}, \mathbf{p})$ from the previous fragment, and the new $(\mathbf{x}, \mathbf{m}, \mathbf{p})$ are compared with those for the first fragment using the technique just described. The common ones are marked for deletion, and the lists of the $(\mathbf{x}, \mathbf{m}, \mathbf{p})$ are joined. The surviving $(\mathbf{x}, \mathbf{m}, \mathbf{p})$ are then consistently and consecutively renumbered. Finally, a Fortran program similar to `bemsa*.f90` is written for

the new combined fragment. For any subsequent fragments, we start with the new combined fragment and then step (b) is repeated until all of the fragments have been added.

In the pairwise method, step (a) is performed and then c) the second fragment is added, augmenting the numberings of the $(\mathbf{x}, \mathbf{m}, \mathbf{p})$. The pair of fragments is examined for common $(\mathbf{x}, \mathbf{m}, \mathbf{p})$, which are marked for deletion. d) the next fragment is similarly added and its $(\mathbf{x}, \mathbf{m}, \mathbf{p})$ are compared in a pairwise fashion with each of the previous fragments, marking duplicates for deletion. After all fragments have been added, the deletions are made and the surviving $(\mathbf{x}, \mathbf{m}, \mathbf{p})$ are consistently and consecutively renumbered. e) the Fortran program similar to `bemsa*.f90` is written for the combined fragments. The sequential and pairwise methods gave identical results for a variety of test cases and the agreement served as a check on our methods.

We notice that for a system made of M fragments, the sequential approach requires M-1 fragment comparisons, while the pairwise method asks for M(M-1)/2. Then, the scaling is linear for the former and polynomial for the latter. However, this advantage for the sequential method in terms of cpu times to construct the fitting basis set may be somehow offset by two aspects. First, the incremental fragment built in the sequential approach gets bigger and bigger while adding new fragments thus requiring more and more comparisons. Secondly, the time needed for deleting duplicates is much shorter than the amount of time (common to both methods) needed to write analytical derivatives in an efficient way, as described next. Overall, then, the two approaches are basically equally effective.

S2. Inputs and Outputs to Mathematica Program

The inputs to the program are as follows: 1) The number of fragments, as well as the number of atoms in the parent, 2) the directory and filename of the `bemsa*.f90` files, one for each of the fragments, 3) a list of the permutational symmetries assumed for the fragments (see below), 4) a list of the atom numbers, following the order of the permutational symmetry (see below), 5) a "data directory" where scratch files may be written (in normal operation,

they are deleted after use) and where the results will be stored, and 6) the directory and filename to which the final Fortran file should be written. More details on steps 3 and 4 will be provided in the examples below.

The outputs of the program are: 1) an analysis of how many **x**, **m**, and **p** were added for each fragment; 2) a test to see a) if all the duplicate polynomials have been eliminated, b) if there is permutational invariance, and c) how much time it takes to create the basis set; 3) a Fortran file written as specified in the input (6).

S3. The A_2BCDEF_2 Example

Here we provide details of two fragmented bases of a molecule of structure A_2BCDEF_2 depicted in Fig. S1 along with the atom numbering we will use to describe it.

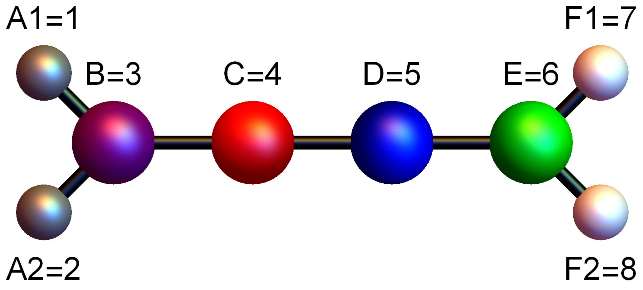

Figure S1: Numbering scheme used for A_2BCDEF_2.

As indicated in Table S1, the symmetry designation for this parent molecule is {2,2,1,1,1,1}. Also shown in the Table are several options for fragmentation of the parent compound along with the symmetry designations of each fragment and an atom numbering scheme that is consistent with the symmetry designation. The right four columns give the number of unique Morse variables, the number of unique monomials, the number of unique polynomials, and the percentage of retained polynomials after elimination of duplicates, as determined by the Mathematica software for the combined fragments and for a maximum polynomial order of 3. We describe below how the Mathematica software arrives at these numbers, while keeping the polynomial order to 3 allows us to determine what is happening without having undue

complications from the much larger number of monomials and polynomials associated with larger orders.

Table S1. Results for fragmentation schemes for A_2BCDEF_2 with maximum 3rd order polynomials. All counts of the number of unique monomials or polynomials include the zeroth-order term.

Fragmentation Scheme	Symmetry Designation	Possible Atom Numbering†	Morse Vars.	No. Mon.	No. Pol.	%Kept Pol.
A_2BCDEF_2 (parent)	{2,2,1,1,1,1}	{1,2,3,4,5,6,7,8}	28	247	1723	100.0
A_2BCD + $CDEF_2$	{2,1,1,1}<br>{2,1,1,1}	{1,2,3,4,5}<br>{7,8,4,5,6}	19	42	332	98.8
A_2BCD + $BCDEF_2$	{2,1,1,1}<br>{2,1,1,1,1}	{1,2,3,4,5}<br>{7,8,3,4,5,6}	22	58	632	96.9
A_2BC + DEF_2	{2,1,1}<br>{2,1,1}	{1,2,3,4}<br>{7,8,5,6}	12	21	99	99.0
A_2BCD + $CDEF_2$ + A_2BEF_2	{2,1,1,1}<br>{2,1,1,1}<br>{2,2,1,1}	{1,2,3,4,5}<br>{7,8,4,5,6}<br>{1,2,7,8,3,6}	28	115	598	95.5

†The numbering listed is one of several possible ones consistent with the symmetry designation; permutation of atoms within a symmetry group as well as reordering of like symmetry groups generates others.

In the first fragmentation scheme we have two fragments each of 5 atoms. The total number of Morse variables for each fragment is given by $(5\times4)/2 = 10$, but we see that one Morse variable, that involving the distance between atoms C and D, is in common between the fragments. The total number of unique Morse variables for the two fragment system is thus 19 rather than 20. The number of monomials for a fragment of {2,1,1,1} symmetry is found from the MSA output[1–3] to be 22, and the number of polynomials is 168, but instead of having 44 monomials and 336 polynomials for the two-fragment system, the Mathematica software shows that there are only 42 unique monomials and 332 unique polynomials. Thus, 2 monomials and 4 polynomials were found to be duplicated between the fragments. It is fairly obvious which monomials were found to be duplicates, since both fragments have a zeroth-order monomial, $m(0) = 1$, and both have the monomial based on the Morse variable with the C-D bond length, in this case $m(10)$ for the first fragment

and $m(8)$ for the second, based on the atom numbering shown in column 3 of the table. In the case of the polynomials, not only do we have the zeroth-order polynomial and first order C-D bond length polynomial in common, but also the second-order and third-order C-D bond length based polynomials, giving altogether 4 polynomials that are duplicated. Thus, the number of unique monomials is 44-2 = 42 and the number of unique polynomials is 336-4 = 332. Note that this fragmentation scheme and maximum polynomial order make the calculation particularly simple, but that with higher orders as well as more and larger fragments with increasing overlap, the situation becomes much less obvious.

The Mathematica software eliminates the duplicate Morse variables, $\mathbf{x}$, monomials, $\mathbf{m}$, and polynomials, $\mathbf{p}$, and provides a simple, recursive scheme for calculating the remaining polynomials similar to that employed in the MSA software, even for very complicated cases. In the sequential build-up scheme, which we will follow here, the first step is to combine the $(\mathbf{x}, \mathbf{m}, \mathbf{p})$ for the first two fragments, augmenting the numbering in the second fragment by the maximum numbers of $(\mathbf{x}, \mathbf{m}, \mathbf{p})$ from the first fragment. To continue the example of the two five-atom fragments considered above, the first fragment has 20 Morse variables, labeled $x(1) - x(20)$, 22 monomials, labeled $m(0) - m(21)$, and 168 polynomials, labeled $p(0) - p(167)$. The combined and augmented numbering has 20 Morse variables, labeled $x(1) - x(20)$, 44 monomials, labeled $m(0) - m(43)$, and 336 polynomials,labeled $p(0) - p(335)$, all taken from the MSA software for each of the two fragments. For example, polynomial $p(20) = p(4) * p(5) - p(18)$ of the second fragment would become $p(167+20) = p(167+4) * p(167+5) - p(167+18)$, or $p(187) = p(171) * p(172) - p(185)$, because the maximum polynomial number in the first fragment is 167.

A sixteen digit random number in the interval between 0.1 and 1.0 is then assigned to the 20 Morse variables, the monomials are evaluated, the results are multiplied by 10^{10}, and the integer parts are taken. Monomials that have the same integer part are marked for deletion. In the current case, monomial $m(22)$ has the same value as monomial $m(0)$ (unity) and $m(24)$ has the same value as $m(1)$ (the value of $x(10)$, the Morse variable based

on the C-D distance). These two m values, $m(22)$ and $m(24)$, are then marked in a way that both indicates that their definitions should be deleted and that other references to them should be replaced by $m(0)$ and $m(10)$, respectively. The polynomials are then evaluated using the same random values for the Morse variables, and, similarly, those that have same integer values are marked for deletion (if they are defining values) or for replacement by their duplicates (if there are references to them). In the current case, there are 28 references to duplicated polynomials and 4 defining values marked for deletion. These four are:

$$\begin{aligned}
&p(168) = p(0) = 1, \\
&p(170) = m(1) = x(10), \\
&p(204) = p(1)p(1) = m(1)m(1) = x(10)^2, \text{ and} \\
&p(330) = p(1)p(204) = p(1)^3 = m(1)^3 = x(10)^3.
\end{aligned} \tag{1}$$

Recalling that $x(10)$ is the Morse variable based on the C-D bond, we see that this gives the same result as we had above, namely that the monomials have 2 duplicates and the polynomials have 4. It remains in the Mathematica software to renumber the monomials and polynomials in sequence and to write a Fortran program file that both incorporates the new definitions and is similar in form to a normal MSA output file. Note that the method by which we have combined the definitions and deleted the duplicates maintains the recursive nature of the calculation, so that the constructions of the final, pruned fitting basis set is still quite efficient.

With more fragments, more overlaps between them, and higher maximum polynomial order, the situation can become more complicated, so that the result cannot be easily anticipated by inspection, as it was in the two five-atom fragment case considered above. In the two-fragment NMA case discussed in the main text, 816 polynomials were eliminated, far too many to keep track of without computational help. In addition, cases with three or more fragments have new properties; some monomials and polynomials might be shared by

just two fragments, and others (such as the corresponding $m(0)$ and $p(0)$ ones) might be shared by all fragments. The method we have developed handles such situations correctly.

The remainder of Table S1 provides some other examples of fragmentation along with the results for the numbers of unique Morse variables, monomials, polynomials, and percentage of kept polynomials.

References

(1) Xie, Z.; Bowman, J. M. Permutationally invariant polynomial basis for molecular energy surface fitting via monomial symmetrization. *J. Chem. Theory Comput.* **2010**, *6*, 26–34.

(2) Original MSA Software. `https://www.mcs.anl.gov/research/projects/msa/`, Accessed: 2019-12-20.

(3) MSA Software with Gradients. `https://github.com/szquchen/MSA-2.0`, Accessed: 2019-01-20.
